# Rupture of the staple suture site after stapling the right inferior pulmonary vein with double rows of staples: a case report

**DOI:** 10.1186/s44215-025-00186-6

**Published:** 2025-01-09

**Authors:** Jun Suzuki, Satoshi Shiono, Hikaru Watanabe, Takayuki Sasage, Kazumasa Hoshijima, Kohei Abe, Tetsuro Uchida

**Affiliations:** Department of Surgery II, Faculty of Medicine, Yamagata University, Yamagata, Japan

**Keywords:** Pulmonary vein stapling, Thoracic surgery complications, Vascular stapler, Postoperative bleeding, Lobectomy, Myocardial cell presence

## Abstract

**Background:**

With advancements in minimally invasive thoracic surgery techniques, such as video-assisted thoracoscopic surgery and robotic surgery, the design of vascular staplers has evolved to meet the requirements of these procedures. Consequently, newer generations of automatic staplers with improved handling and reduced size have been introduced, such as two-row staplers, which are more maneuverable and less bulky than their three-row counterparts.

**Case presentation:**

A 68-year-old man with lung cancer underwent a right middle and lower lobectomy due to tumor invasion into the central middle bronchial trunk, rendering the preservation of the middle lobe impossible. His medical history included chronic atrial fibrillation. The surgery involved a posterolateral incision and a fifth intercostal thoracotomy, where various pulmonary arteries and veins were dissected using vascular staples. Despite completing the surgery without initial complications, the patient experienced significant postoperative bleeding, leading to approximately 800 mL of bloody fluid being drained after coughing episodes. Reoperation was necessary to address and control the bleeding, which was challenging due to the location and nature of the hemorrhage. The source was identified at the transected edge of the inferior pulmonary vein, requiring direct suture after pericardium incision for better access. The total operative time amounted to 751 min, with a blood loss of 2092 mL. The patient recovered smoothly from the second operation and was discharged on the fifth postoperative day. Histopathological examination revealed myocardial cell presence adjacent to the pulmonary vein wall, suggesting that vein thickening could have played a role in the observed postoperative bleeding.

**Conclusions:**

In conclusion, when selecting staples for vascular use, particularly for the detachment of pulmonary veins, it is advisable to carefully choose between two-row and three-row staples.

**Supplementary Information:**

The online version contains supplementary material available at 10.1186/s44215-025-00186-6.

## Background

In the field of thoracic surgery, stapling of pulmonary vessels is widespread. With advancements in minimally invasive thoracic surgery techniques, such as video-assisted thoracoscopic surgery and robotic surgery, the design of vascular staplers has evolved to meet the demands of these procedures. Consequently, newer generations of automatic staplers with improved handling and reduced size have been introduced, such as two-row staplers, which are more maneuverable and less bulky than their three-row counterparts.

These two-row staplers have become increasingly popular in thoracic surgery owing to their improved maneuverability and versatility, making them suitable for use in confined spaces during video-assisted thoracoscopic surgery and robotic surgery. Additionally, the reduced size of these staplers allows for more precise placement of staples. However, the closure of vessels with double-row staples may be lesser than with triple-row staples, and their use should be carefully considered depending on the size of the vessel.

The number of double staple rows, with thinner shafts and easier staple manipulation in tight spaces, is expected to increase. However, stapling failure and staple-related complications can occur, and staple-induced hemothorax has been reported [1, 2]. It has been suggested that lifting or twisting the staples while stapling blood vessels may put unexpected stress on the blood vessels to be transected, potentially resulting in bleeding [3]. Considering most reports on thoracic surgery have tended to focus on pulmonary artery procedures, we report a case in which a pulmonary vein procedure caused major bleeding immediately after the procedure, resulting in reoperation.

## Case presentation

**Fig. 1:**
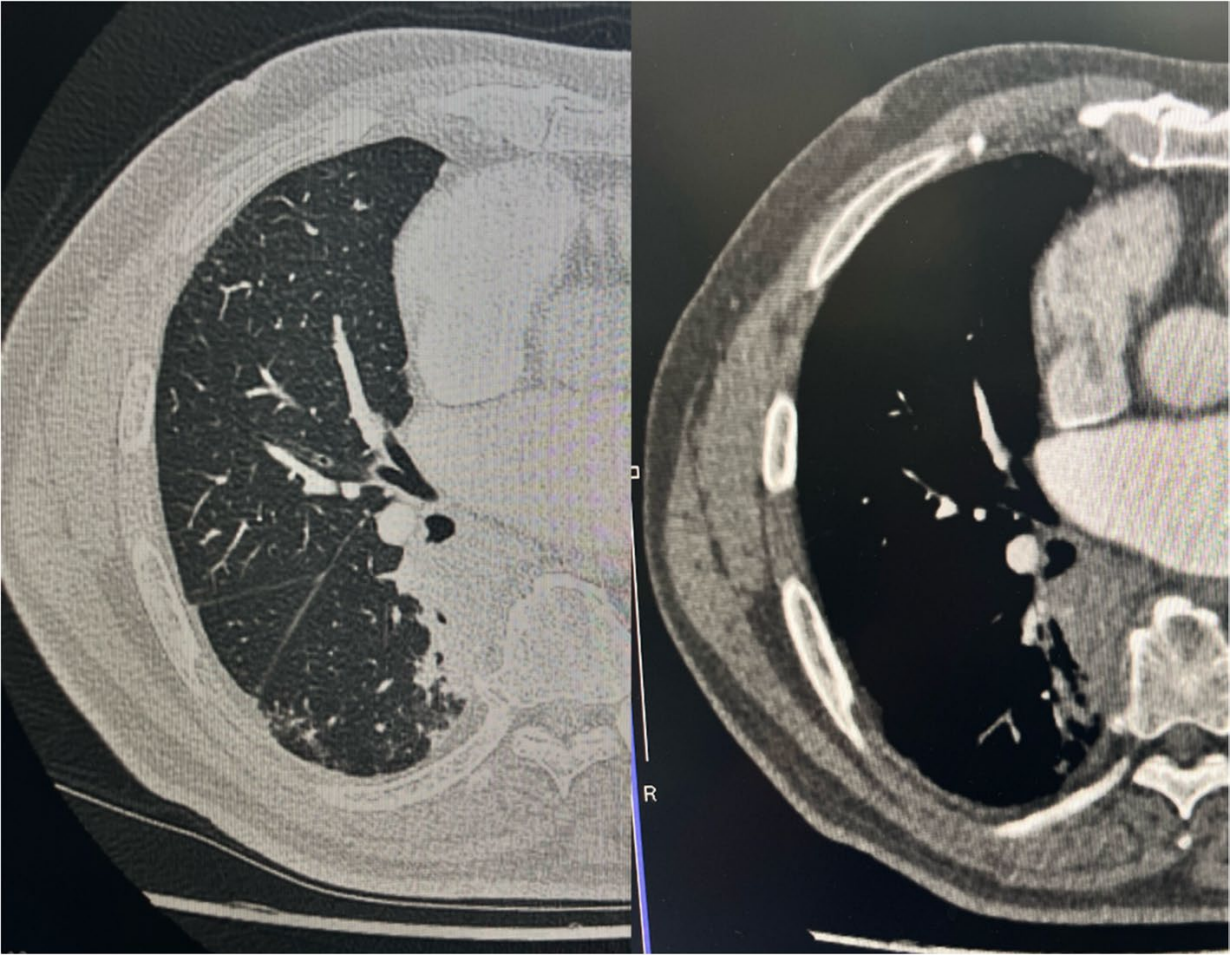
The computed tomography scan showed a tumor in the right lower lobe, measuring 43.0 × 40.0 × 36.0 mm. The cephalic side of the tumor extended to the middle lobar bronchus

A 68-year-old man with lung cancer underwent a right middle and lower lobectomy after a tumor in the lower lobe had slightly invaded proximal to the hilum. Preservation of the middle lobe was not possible (Fig. 1). His medical history included chronic atrial fibrillation, and preoperative echocardiography revealed that the left atrial diameter had enlarged to 46 mm. He had been taking edoxaban tosylate hydrate for the prevention of cerebral infarction but discontinued the medication 2 days prior to surgery. Curative surgery was performed using posterolateral incision and fifth intercostal thoracotomy. Upon examining the thoracic cavity, fortunately, no evident adhesion between the tumor and the mediastinum was observed. The lower lobe superior segmental pulmonary artery (A6) and common basilar pulmonary artery were dissected with double rows of vascular staples (ECHELON FLEX™ Powered Vascular Stapler Ethicon Endo-Surgery, Cincinnati, OH, USA). Next, there were two middle lobe pulmonary arteries (A4 and A5), and each was double-ligated at the proximal side with 2-0 silk sutures and then divided.

The middle lobe pulmonary vein (V4 + 5) was divided with double rows of vascular staples (ECHELON FLEX™ Powered Vascular Stapler Ethicon Endo-Surgery, Cincinnati, OH, USA). As the final step of vascular management, the inferior pulmonary vein was divided. Although the left atrial diameter was dilated due to chronic atrial fibrillation, it was determined that division with a double-row vascular stapler, as in other areas, was feasible, and the vessel was divided with double rows of vascular staples (ECHELON FLEX™ Powered Vascular Stapler Ethicon Endo-Surgery, Cincinnati, OH, USA) (Supplementary Video S1).

Finally, the bronchus was dissected using triple-row staplers (Ethicon Endo-Surgery, Cincinnati, OH, USA) with a green cartridge. Surgery was completed without any further complications. Post-surgery, chest radiography confirmed no problems, and awakening anesthesia was attempted.

However, after the patient coughed several times, approximately about 800 mL of bloody fluid was transiently drained from the chest drain, and swelling at the wound site was observed, suggesting that intrathoracic pressure was increasing due to bleeding. The drainage was stopped thereafter; however, the decision was made to reopen the chest to check the bleeding site and achieve complete hemostasis.

**Fig. 2:**
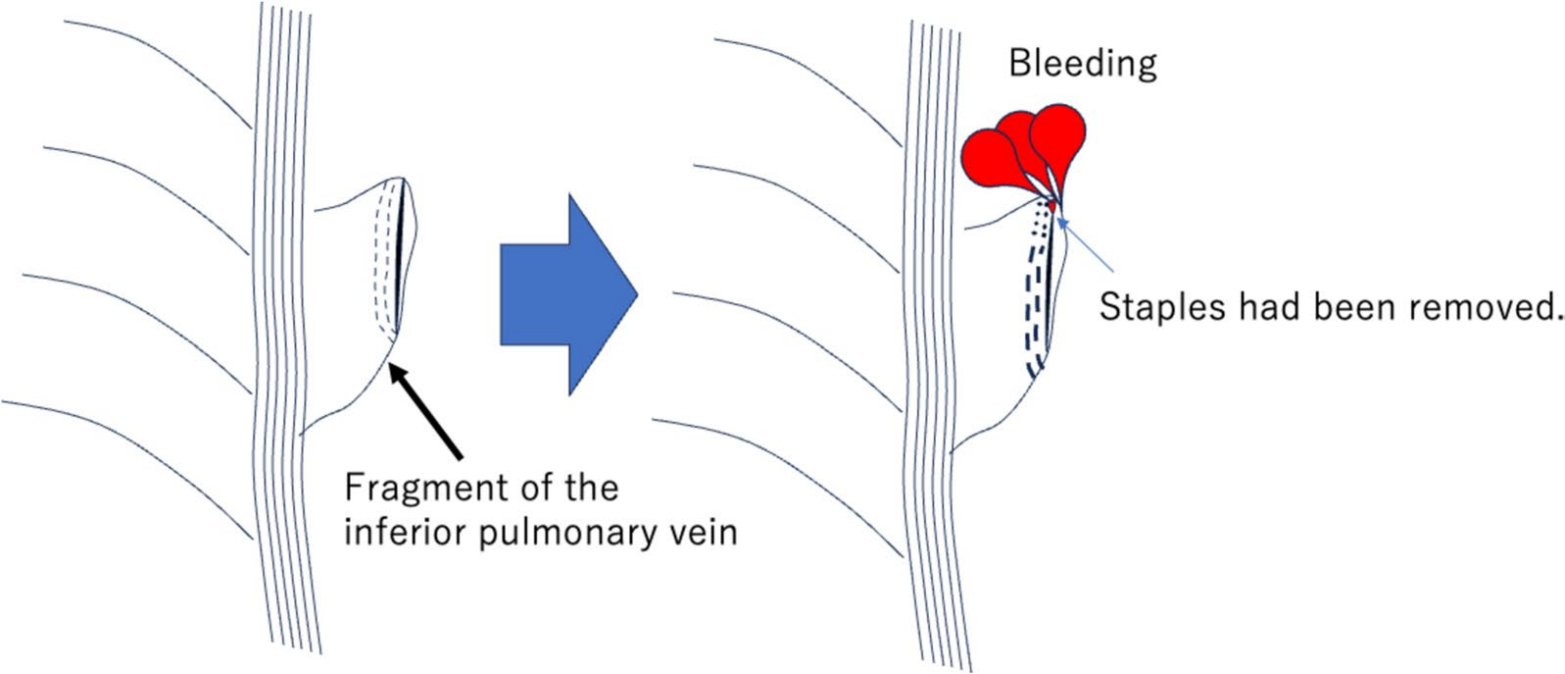
Schema for rebleeding from the inferior pulmonary vein transection site

The thoracic cavity was filled with a hematoma; after its removal and search for its source, minor bleeding was observed at the upper edge of the transected inferior pulmonary vein. A few staples were removed. At that point, the hemorrhage subsided; however, the abrasion of the transected edge caused the hemorrhage to erupt (Fig. 2) (Supplementary Video S2). Bleeding was difficult to control using compression and fibrin sealant patches. Furthermore, it was also difficult to lift and directly suture the dissected fragment. Therefore, the pericardium was incised, and the procedure was performed in the pericardium. Vascular forceps were used to block the central portion of the dissected fragment in the lower right pulmonary vein. Suture closure was performed with a 7–0 monofilament (7–0 Prolene® ETHICON, Johnson. & Johnson, Tokyo, Japan). The total operative time for all procedures was 751 min, and the total blood loss was 2092 mL.

**Fig. 3:**
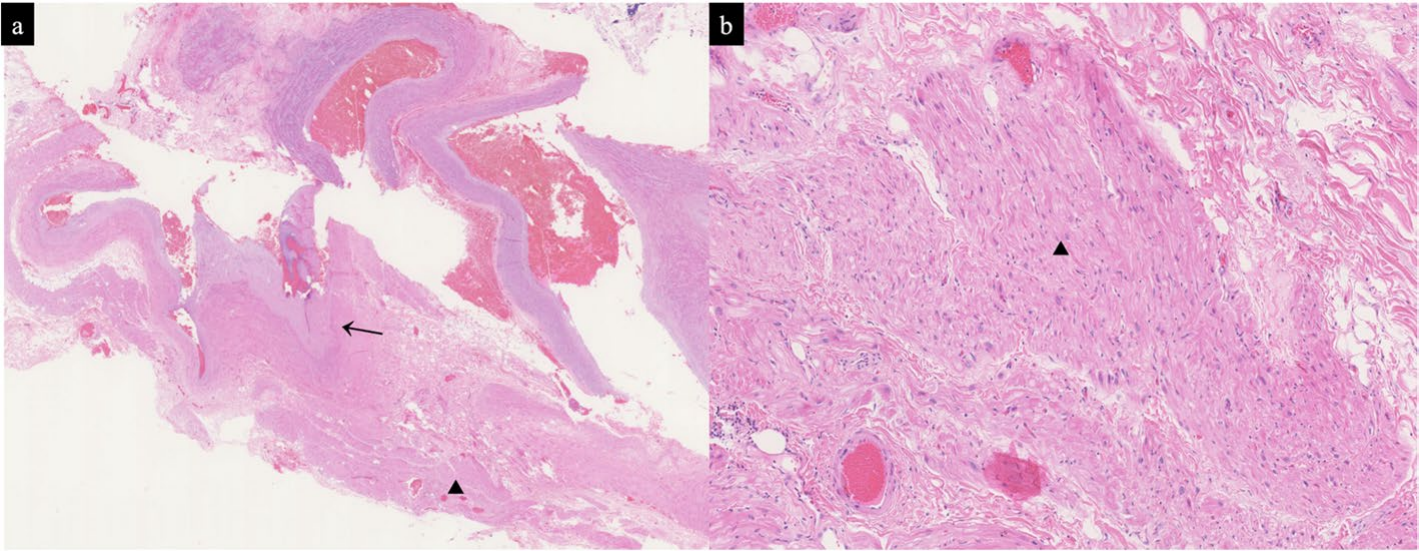
Pathological findings of the vein stump. The arrows ( ←) indicate the vessel wall, and the triangles (▲) indicate the adjacent myocardial cells. **a** is at low magnification, and **b** is at high magnification of the myocardium

The postoperative course was uneventful, and the patient was discharged on the fifth postoperative day after the second operation. The final pathological diagnosis was keratinizing squamous cell carcinoma, with the maximum tumor size measuring 3.0 cm. There was no lymph node metastasis, and the pathological stage was T1cN0M0-1A3 (according to the 8th edition of the TNM classification). In the pathological findings of the inferior pulmonary vein stump, myocardial cells were observed near the vessel wall, suggesting the possibility of thickening due to this finding (Fig. 3).

## Discussion and conclusions

Vascular staplers have become indispensable tools in thoracic surgery and facilitate the creation of anastomoses, with numerous advantages [4, 5]. A benefit of vascular staplers is that they significantly reduce the time required to create an anastomosis. Another crucial advantage is that they can help reduce the risk of bleeding during and after surgery, particularly in fragile or difficult-to-access vessels. By minimizing bleeding, vascular staplers can increase the safety and reduce the invasiveness of surgery [6, 7].

Shimizu et al. [3] reported that adverse reactions due to pulmonary vascular stapling occurred in 1.19% of patients who underwent lung surgery. Yano et al. [4] reported the adverse events (AEs) following pulmonary vascular stapling in a multi-institutional retrospective study. Their analysis revealed that nine AEs were judged to be related to stapling in 3393 vascular staplings (0.27%), and AEs in the pulmonary artery were observed more frequently than those in the pulmonary vein for intraoperative stapling. In the present case, the inferior pulmonary vein was dissected using two rows of staples. However, immediately before the patient awakened from anesthesia, bleeding occurred at the unstapled part of the inferior pulmonary vein transection site. We did not intentionally centralize the line of dissection of the inferior pulmonary vein, nor did we intend to dissect it at any twisted or unstable angle. Even if the dissecting line is not very central, thickening of the pulmonary vein wall can occur due to unexpected entry of myocardial tissue into the dissecting wall of the pulmonary vein.

The pulmonary veins shifted into the myocardium as they moved toward the center. Pericardial thickness also increased. In such cases, two rows of staples may not be sufficient for sealing.

A limitation of this study is that it cannot explain the criteria that would allow us to specify a clear distinction between using two or three rows of staples. Further, it is unclear whether specifications for three rows of staples would prevent bleeding.

In conclusion, when selecting staples for vascular use, particularly for the detachment of pulmonary veins, it is advisable to carefully choose between two-row and three-row staples. While the precise impact of pulmonary vein wall thickening and myocardial cell presence on postoperative bleeding remains undetermined, this case underscores the need for vigilance regarding these factors when using vascular staplers. Surgeons should consider the possibility of such anatomical variations to mitigate postoperative bleeding risks.

## Supplementary Information

Supplementary Material 1: Supplementary video S1. Dissection of the inferior pulmonary vein using two rows of staples.

Supplementary Material 2: Supplementary video S2. Visuals after the abrasion of the transected edge caused the hemorrhage to erupt.

## Data Availability

The case report and patient consent form are available from the corresponding author upon reasonable request.

## Abbreviation

AE: Adverse event
